# Salt-Sensitivity of Blood Pressure and Insulin Resistance

**DOI:** 10.3389/fphys.2021.793924

**Published:** 2021-12-13

**Authors:** Lale A. Ertuglu, Fernando Elijovich, Cheryl L. Laffer, Annet Kirabo

**Affiliations:** ^1^Division of Nephrology, Department of Medicine, Vanderbilt University Medical Center, Nashville, TN, United States; ^2^Division of Clinical Pharmacology, Department of Medicine, Vanderbilt University Medical Center, Nashville, TN, United States

**Keywords:** salt-sensitivity, blood pressure, insulin resistance, immune activation, PPARγ

## Abstract

Salt sensitivity of blood pressure (SSBP) is an independent risk factor for cardiovascular morbidity and mortality that is seen in both hypertensive and normotensive populations. Insulin resistance (IR) strongly correlates with SSBP and affects nearly 50% of salt sensitive people. While the precise mechanism by which IR and SSBP relate remains elusive, several common pathways are involved in the genesis of both processes, including vascular dysfunction and immune activation. Vascular dysfunction associated with insulin resistance is characterized by loss of nitric oxide (NO)-mediated vasodilation and heightened endothelin-1 induced vasoconstriction, as well as capillary rarefaction. It manifests with increased blood pressure (BP) in salt sensitive murine models. Another common denominator in the pathogenesis of insulin resistance, hypertension, and salt sensitivity (SS) is immune activation involving pro-inflammatory cytokines like tumor necrosis factor (TNF)-α, IL-1β, and IL-6. In the last decade, a new understanding of interstitial sodium storage in tissues such as skin and muscle has revolutionized traditional concepts of body sodium handling and pathogenesis of SS. We have shown that interstitial Na^+^ can trigger a T cell mediated inflammatory response through formation of isolevuglandin protein adducts in antigen presenting cells (APCs), and that this response is implicated in salt sensitive hypertension. The peroxisome proliferator-activated receptor γ (PPARγ) is a transcription factor that modulates both insulin sensitivity and BP. PPARγ agonists increase insulin sensitivity and ameliorate salt sensitivity, whereas deficiency of PPARγ results in severe insulin resistance and hypertension. These findings suggest that PPARγ plays a role in the common pathogenesis of insulin sensitivity and salt sensitivity, perhaps *via* effects on the immune system and vascular function. The goal of this review is to discuss those mechanisms that may play a role in both SSBP and in insulin resistance.

## Introduction

Salt sensitivity (SS) of blood pressure (BP) is a phenotype characterized by changes of BP that parallel changes in dietary salt intake. SS affects more than half of all hypertensive subjects as well as a quarter of normotensive individuals in the United States (Weinberger et al., 1986) and is a cardiovascular risk factor for both normotensive and hypertensive humans (Morimoto et al., 1997; Weinberger et al., 2001). As a polygenic trait normally distributed in human populations, SS is well-known to be associated with insulin resistance (IR; Zavaroni et al., 1995; Fuenmayor et al., 1998; Suzuki et al., 2000; Giner et al., 2001). In fact, epidemiological studies suggest that approximately 50% of salt sensitive individuals are insulin resistant (Reaven, 2003), independent of confounding factors such as age, obesity, and glucose intolerance (Galletti et al., 1997). Furthermore, the degree of salt sensitivity seems to correlate with the severity of IR (Zavaroni et al., 1995; Giner et al., 2001), suggesting a causal relationship between the two states. However, the mechanisms underlying this association remain unclear. This article reviews the existing evidence on the interplay between salt sensitivity and insulin resistance and propose mechanisms to explain their relationship.

## Pathogenesis of Salt Sensitivity

Since SSBP was described, its underlying pathophysiologic mechanisms have been a matter of controversy. The traditional view follows the classic concept of Guyton (1991), who postulated that SS individuals must have an intrinsic defect in renal sodium handling. According to this view, a salt load expands plasma volume to reach isosmotic balance, which in turn activates systemic and renal natriuretic mechanisms, leading to renal salt excretion without change in arterial pressure (Guyton, 1991). Thus, only an impairment in some renal natriuretic system could lead to hypertension. While defects in renin angiotensin system (Giner et al., 2000), renal sodium transport (Lluch et al., 1996), and sympathetic system (de la Sierra et al., 1996) among others, have been implicated, the exact pathogenesis has not been established. Impaired natriuresis should induce salt retention and plasma volume expansion in SS individuals. However, several studies showed that there is no difference in sodium balance, plasma volume, and cardiac output in response to salt loading or depletion between SS and salt resistant (SR) individuals (Schmidlin et al., 2007; Laffer et al., 2016). Instead, hemodynamic measurements in humans and animals revealed an absence of the normal vasodilator response to salt or even a paradoxical vasoconstriction in SS (Ganguli et al., 1979; Sullivan and Ratts, 1983; Simchon et al., 1989; Qi et al., 1999; Schmidlin et al., 2007; Laffer et al., 2016). This would imply a shift of the paradigm from one of renal excretory defects to one of extrarenal mechanisms producing vascular dysfunction. Indeed, while cardiac output increases after a salt load in both SR and SS subjects, SS subjects lack the concomitant decrease in total peripheral resistance (TPR) that is seen in SR subjects. Similarly, TPR is also unchanged after salt depletion in SS subjects. These findings suggested that BP elevation in SS was mediated by abnormalities in vasoconstrictor/vasodilator responses.

Our understanding of whole-body Na^+^ distribution has been recently expanded by new knowledge about the storage of Na^+^ in the interstitial compartment. Previous literature on salt sensitivity regarded body salt balance in terms of the traditional isoosmolar sodium distribution, including the intravascular, interstitial, and intracellular compartments. However, recent studies showed that Na^+^ may accumulate in the interstitium without commensurate water retention but in association with glycosaminoglycans instead (Titze et al., 2004; Wenstedt et al., 2021). Whether this Na^+^ is or is not hyperosmolar is controversial (Rossitto et al., 2020), but irrelevant in terms of activation of immune cells, which is due to the Na^+^ concentration, not to its osmolality (Barbaro et al., 2017). In any case, the finding sheds doubt on the traditional model of sodium-water balance (Machnik et al., 2009; Wiig et al., 2018). In murine models, extrusion of Na^+^ from this compartment involves a macrophage salt-sensitive tonicity-responsive enhancer binding protein (TonEBP) and stimulation of vascular endothelial growth factor C (VEGFc) leading to lymphangiogenesis (Machnik et al., 2010; Wiig et al., 2013). Blocking this pathway by pharmacological or genetic means results in salt-sensitive hypertension.

Studies using ^23^Na MRI confirmed that Na^+^ is stored in the interstitia of skin and skeletal muscle in humans, indicating that this may also occur in other organs less accessible for measurement, and that this storage may provide a buffering system for excess salt intake (Kopp et al., 2013). Others have shown that skin Na^+^ positively correlates with BP. We have made a similar observation with muscle Na^+^, which positively correlated with systolic and diastolic blood pressures in patients studied either on their usual diets or during the sodium loading and depletion phases of a research protocol (Sahinoz et al., 2021). Also, skin and skeletal muscle Na^+^ increase with aging and hypertension (Rossitto et al., 2020), suggesting a direct role of these stores in BP regulation. Some evidence for differential regulation of this Na^+^ storage between SS and SR individuals has been obtained (Laffer et al., 2016), but this remains to be established with certainty in future studies.

## Insulin Resistance, Hypertension, and Salt Sensitivity

Insulin resistance, or reduced insulin sensitivity, is a key component of the metabolic syndrome, which includes hypertension, obesity, and dyslipidemia. Research in the last three decades has proved a strong association between hypertension and insulin resistance, and this relationship is stronger in salt sensitive hypertension (Yatabe et al., 2010).

Salt intake has a close relationship with hypertension (Grillo et al., 2019) and may be a determinant of the pathogenetic link between salt sensitivity and insulin resistance, because it impairs insulin sensitivity in normotensive and hypertensive patients with salt sensitivity but not in those with salt resistance (Sharma et al., 1991; Zavaroni et al., 1995). A high-salt diet exaggerated the insulin response to an oral glucose load in SS but not SR patients (Ferri et al., 1998; Fuenmayor et al., 1998). This suggests that in salt sensitive states, high salt intake may exacerbate insulin resistance. In turn, insulin resistance seems to heighten the blood pressure response to sodium intake (Zhou et al., 2014). Whether the effect of aldosterone, many times inappropriately secreted in SS subjects, plays a role in determining insulin resistance *via* hybridization of the insulin and insulin growth factor receptors (Sherajee et al., 2012) is not known.

Several mechanisms have been postulated to explain the association between insulin resistance, salt sensitivity, and hypertension. In the following sections, we will describe renal vascular and immune mechanisms, including novel findings about a role for endothelin (Liu et al., 2013) and for interstitial Na^+^ storage (Nikpey et al., 2017).

### Renal Sodium Retention

**Figure 1:**
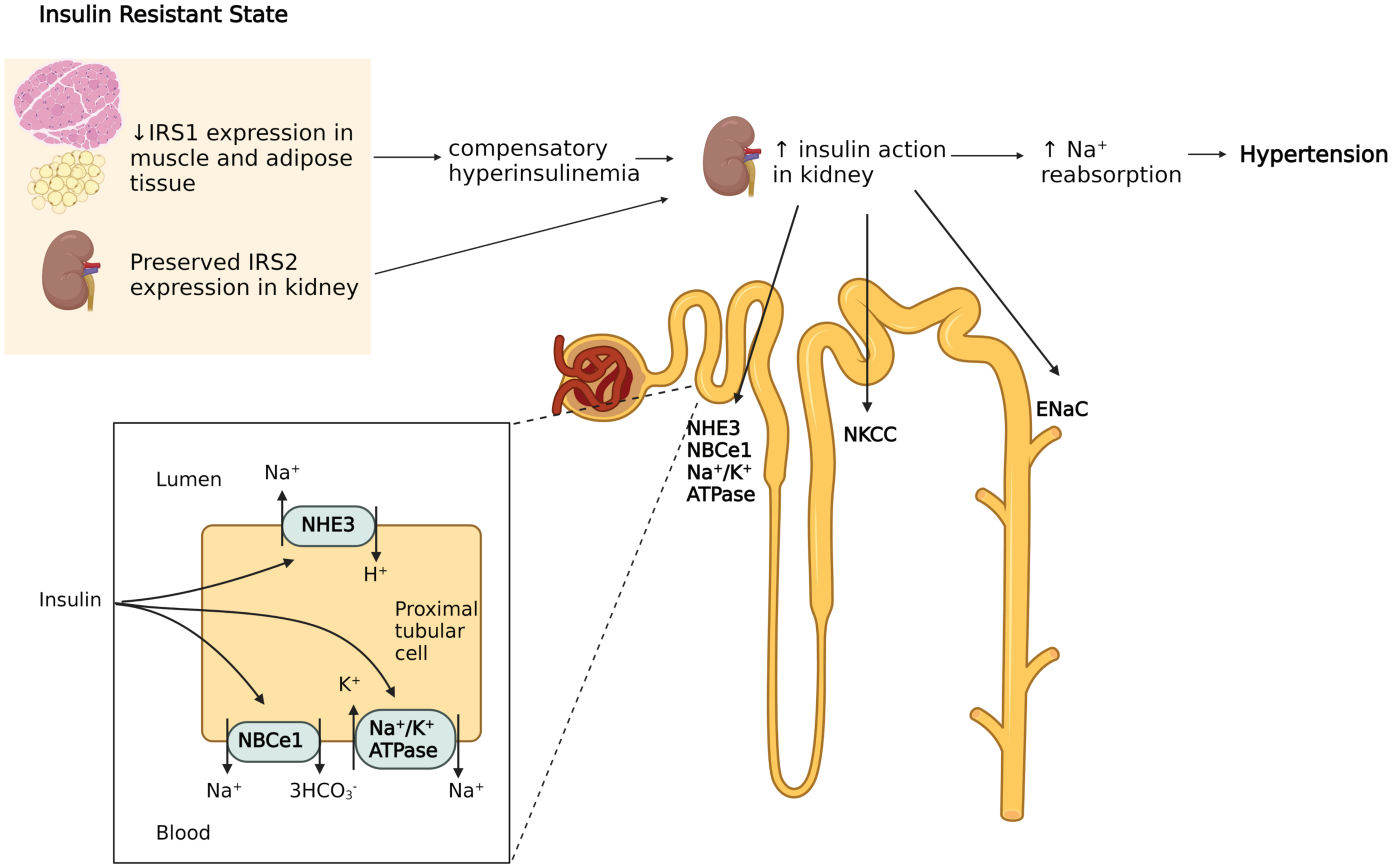
Hyperinsulinemia induced sodium retention in the kidney. Insulin resistance (IR) in humans selectively affects insulin receptor substrate (IRS) 1 in muscle and adipose tissue, while the function of IRS2 in kidney is preserved. The compensatory hyperinsulinemia of insulin resistant states increases insulin’s effects in renal tubules by activating of Na^+^/H^+^ exchanger type 3, Na^+^/K^+^ ATPase, Na-K-2CL cotransporter, sodium-bicarbonate cotransporter (NBCe1) and the epithelial sodium channel (ENaC). This leads to increased sodium retention which contributes to hypertension. The figure created with BioRender.com.

Insulin resistance is primarily due to downregulation or inactivation of insulin signaling, which is mainly mediated by insulin receptor substrates (IRSs) in effector organs. IRSs exhibit tissue specific distribution. Whereas IRS1 facilitates insulin dependent glucose transport in adipose and skeletal muscle tissue, IRS2 primarily mediates the effects of insulin on the kidney (Kido et al., 2000; Hookham et al., 2013). The latter increase Na^+^ reabsorption in multiple nephron segments *via* stimulation of Na^+^/H^+^ exchanger type 3 (Klisic et al., 2002), Na^+^/K^+^ ATPase (Ewart and Klip, 1995), Na-K-2CL cotransporter (Tsimaratos et al., 2003; Horita et al., 2011), sodium-bicarbonate cotransporter (NBCe1; Horita et al., 2011; Pavlov et al., 2013), and the epithelial sodium channel (ENaC; Loffing and Korbmacher, 2009). It is now known that insulin resistance affects IRSs’ expression differently in each target tissue (Soleimani, 2015). For example, insulin resistant humans have significantly reduced IRS1 and IRS2 expression in metabolically active tissues such as muscle, while IRS2 expression in the kidney is exceptionally preserved (Sechi, 1999; Nakamura et al., 2015). Thus, the compensatory hyperinsulinemia required to maintain normoglycemia can aggravate insulin sodium-retaining actions (Miyazaki et al., 1996), which may potentially lead to hypertension (Figure 1). This is supported by the observation that mice lacking the insulin receptor specifically in the collecting duct principal cells showed significantly lower ENaC activity, while ENaC subunit expression was not changed compared to wild type mice. Also, wild type mice had enhancement in ENaC activity following insulin treatment, indicating insulin mediated channel opening in renal tubules (Pavlov et al., 2013). Thus, selective insulin resistance, through decreasing sodium excretion, may increase the effective sodium load in response to high oral sodium intake, which could aggravate the blood pressure response in salt sensitive patients.

**Figure 2:**
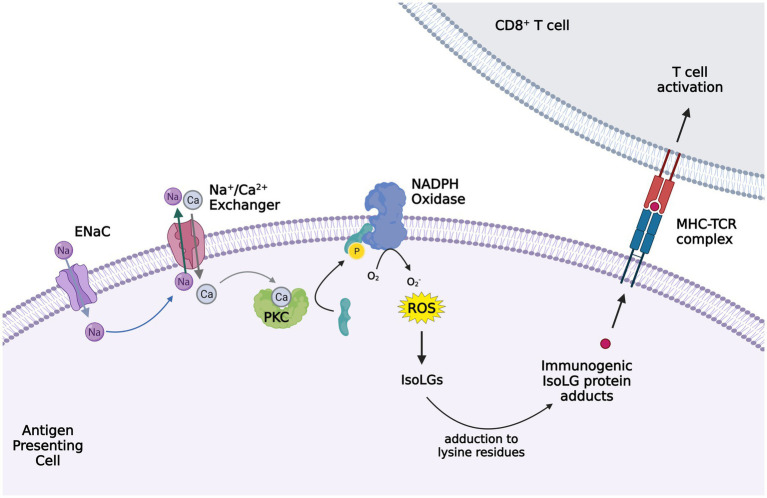
Activation of ENaC in antigen presenting cells (APC) leading to isoleuvoglandin (IsoLG) formation and activation of CD8^+^ T cells. Elevated interstitial sodium (Na^+^) enters APCs, in particular dendritic cells, through ENaC and triggers intracellular calcium entry *via* the sodium calcium exchanger. The increased intracellular calcium activates protein kinase C (PKC), which in turn activates NAPDH oxidase *via* phosphorylation of p47*phox*. Superoxide and ROS formation by NAPDH oxidase leads to IsoLG production, which adduct to the lysine residues in proteins and form IsoLG adducts that are presented as neoantigens and trigger CD8^+^ T cells activation. The figure created with BioRender.com.

**Figure 3:**
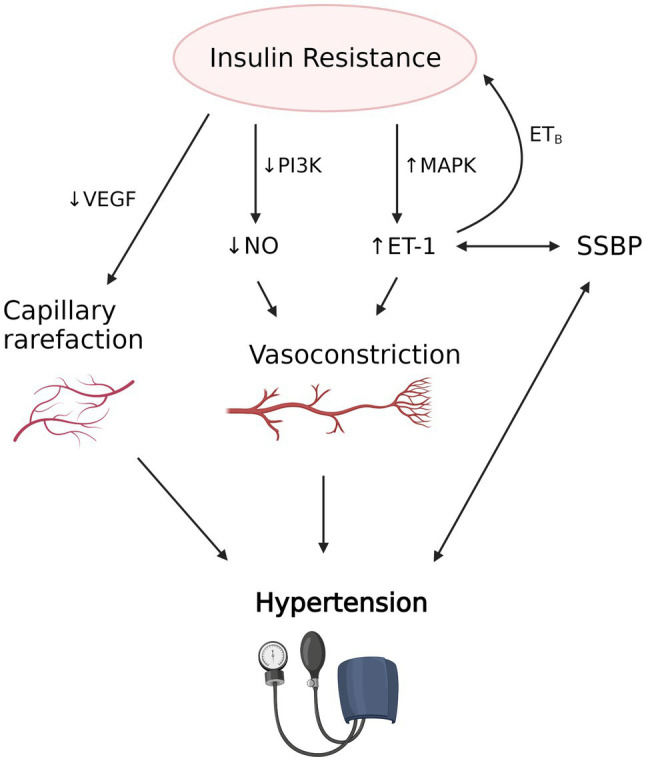
The proposed interplay between insulin resistance induced vascular dysfunction, salt sensitivity of blood pressure (SSBP) and hypertension. Insulin resistance is associated with decreased nitric oxide (NO) production due to decreased activity of PI3K and increased endothelin-1 (ET-1) production due to increased mitogen-activated protein kinase (MAPK) activity, which results in vasoconstriction in precapillary arterioles. Decreased vascular endothelial growth factor (VEGF) and angiogenesis also causes capillary rarefaction. Evidence suggests that SSBP, which strongly correlates with hypertension, is associated with increased availability of ET-1. Increased ET-1, in turn, may contribute to the development of insulin resistance *via* the action of ET_B_ receptor in adipose tissue. Vascular dysfunction caused by insulin resistance and possibly SSBP leads to hypertension and its clinical manifestations. The figure created with BioRender.com.

**Figure 4:**
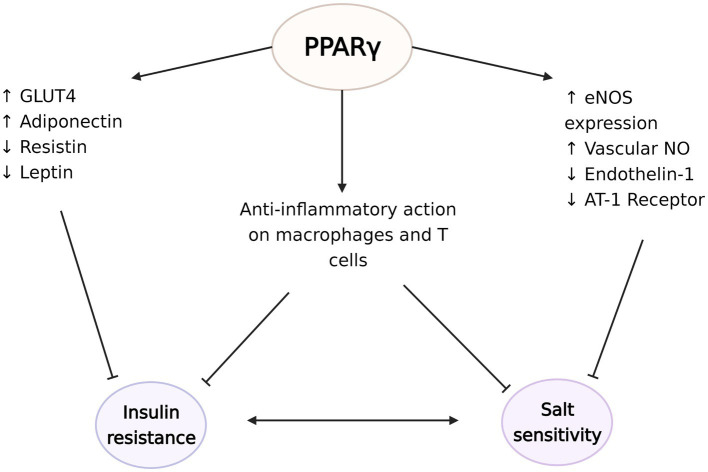
The protective effects of peroxisome proliferator-activated receptor γ (PPARγ) against the development of insulin resistance and salt sensitivity. PPARγ plays a critical role in regulation of the expression of glucose transporter type 4 (GLUT4), adiponectin, resistin, and leptin to increase insulin sensitivity. At the same time, PPARγ induces endothelial nitric oxide synthase (eNOS) expression and NO synthesis and decreases endothelin-1 and angiotensin II type 1 (AT-1) receptor availabilities. These vasodilatory actions potentially ameliorate the vascular dysfunction seen in salt sensitivity. PPARγ activation also has anti-inflammatory effects on macrophages and regulatory T cells in the visceral adipose tissue, which possibly counteract the development of insulin resistance and salt sensitivity. The figure created with BioRender.com.

### Vascular Dysfunction

Another mechanism by which IR is postulated to induce hypertension is through vascular insulin resistance (Yki-Järvinen and Utriainen, 1998). Insulin has important vasodilatory effects in skeletal muscle to augment muscle blood flow and glucose transport (Steinberg et al., 1996) and these are impaired in insulin resistant states (Mather et al., 2013). Insulin relaxes precapillary arterioles to facilitate transcapillary insulin transport, increasing microvascular blood flow by a nitric oxide (NO) dependent process termed microvascular recruitment (Herkner et al., 2003; Vincent et al., 2004). Insulin-induced NO production is due to activation of nitric oxide synthase *via* the phosphatidylinositol 3-kinase (PI3K) pathway, similar to other metabolic insulin signaling (Muniyappa et al., 2020).

In contrast to its vasodilatory effects, insulin also triggers vasoconstriction *via* mitogen-activated protein kinase (MAPK)-dependent production of vasoconstrictor endothelin 1 (ET-1). This mechanism is involved in fine-tuning of vascular tone (Muniyappa et al., 2020). Selective resistance to the action of insulin on the PI3K-dependent pathway depresses nitric oxide synthesis, whereas the unaffected MAPK pathway maintains endothelin-1 production. This results in a shift of the normally delicate balance between the two opposing vascular actions of insulin toward vasoconstriction and hypertension (Ko et al., 2010). Supporting the involvement of insulin resistance-associated vascular dysfunction in SSBP are observations in Dahl salt sensitive rats, which after a high salt diet exhibit hypertension, metabolic insulin resistance, impaired insulin-dependent activation of PI3K/endothelial NO synthase (eNOS), and impaired NO-mediated vasorelaxation (Zhou et al., 2009, 2010). Other studies of mice have also found high salt diet induced reduction in NO synthesis, through Rho kinase-dependent inhibitory phosphorylation of eNOS by circulating interleukin-17 (IL-17; Faraco et al., 2018).

Insulin’s vasodilatory effects are also crucial to ensure adequate transendothelial transport, a rate limiting step of insulin’s metabolic action (Yang et al., 1989). There is evidence that microvascular IR precedes metabolic IR (Zhao et al., 2015), suggesting an important role for endothelial dysfunction in the pathogenesis of metabolic IR. IR also causes capillary rarefaction in the skeletal muscle, a process of reduced capillary density due to impairment in the angiogenesis mediated by the vascular endothelial growth factor (VEGF; Chung et al., 2006; Frisbee, 2007). This contributes further to insulin-induced vascular dysfunction.

The evidence above suggests that vascular dysfunction induced by insulin resistance may play a role in the pathogenesis of hypertension. Conversely, hypertension associated increased vascular resistance may contribute to the development of metabolic insulin resistance. Indeed, a relationship between hypertension and insulin resistance is further suggested by the finding that monotherapies with either enalapril (vasodilator ACE inhibitor) or rosiglitazone (insulin sensitizer), effectively reduce metabolic insulin resistance, plasma levels of ET-1 and blood pressure in spontaneously hypertensive rats (Potenza et al., 2006). Similar trends have also been reported in nondiabetic hypertensive patients treated with insulin sensitizers and vasodilator antihypertensive agents (Raji et al., 2003; Geng et al., 2013).

## Interplay Between Endothelin-1 and Ir in Hypertension

Endothelin-1 is a potent vasoconstrictor secreted by vascular endothelial cells and has been long thought to play a role in the development of the hypertension component of the metabolic syndrome (Dhaun et al., 2008). As discussed above, selective insulin resistance enhances the prohypertensive action of ET-1 by disrupting its physiological balance with NO. This is supported by observations in rodents and humans. In rats, a continuous insulin infusion leads to development of insulin resistance and hypertension along with higher plasma ET-1 levels (Juan et al., 2004). In humans, (a) insulin stimulates ET-1 production during an euglycemic hyperinsulinemic clamp, (b) insulin resistant patients have elevated plasma ET-1 concentrations (Wolpert et al., 1993), (c) plasma insulin and ET-1 levels correlate in essential hypertensive patients (Zaporowska-Stachowiak et al., 1997), and (d) a mixed ET-1 type A (ET_A_) and type B (ET_B_) receptor antagonist produces vasodilation in insulin resistant but not insulin sensitive humans (Shemyakin et al., 2006). All these findings suggest a role of insulin-induced ET-1 predominance in the development of hypertension.

Conversely, ET-1 may play a causal role in the development of insulin resistance, since it inhibits insulin mediated glucose uptake in adipocytes through ET_B_ receptors (Chou et al., 1994). It is *via* stimulation of this receptor that ET-1 regulates adiponectin expression and promotes adipose tissue deposition and insulin resistance (Juan et al., 2007; Rivera-Gonzalez et al., 2020).

## Interplay Between Endothelin-1 and Salt in Hypertension

Murine studies have also shown that high salt intake upregulates vascular ET-1 expression independent of changes in blood pressure (Tsai et al., 2006). Also, if the endothelin-1 gene is knocked out from the endothelium (the VEET KO mouse), a high salt diet fails to increase mean arterial pressure to the same extent as in control mice (Speed et al., 2015).

In normotensive and hypertensive humans, high salt intake increases plasma ET-1 levels (Liu et al., 2013). We have discussed above how tissue Na^+^ storage is involved in salt sensitivity of blood pressure. It is therefore noteworthy that skin Na^+^ accumulation in response to high salt diet is accompanied by increased ET-1 mRNA expression in vascular tissues (Speed et al., 2015). Therefore, ET-1 may modulate the blood pressure response to salt intake through a direct vasoconstrictive effect as well as an effect on tissue Na^+^ accumulation. Actually, the interaction between ET-1 and salt in hypertension may have a third player, because tissue Na^+^ also associates with insulin resistance, with the caveat that the latter observation has only been made in patients with end stage kidney disease (Sahinoz et al., 2020).

Several observations suggest that participation of ET-1 in salt-induced hypertension may be different in SS vs. SR subjects. For example, ET-1 levels are higher in salt sensitive than in salt resistant essential hypertensive patients (Ferri et al., 1998). Also, while receiving an intermediate salt diet, salt sensitive patients have a higher plasma ET-1 response to oral glucose load than their salt resistant counterparts (Ferri et al., 1998). Finally, in a study of 155 normotensive and mild hypertensive patients, a 7-day high salt diet increased plasma ET-1 levels and arterial stiffness much more in SS than in SR patients (Liu et al., 2013). All these studies show that exaggerated ET-1 responses to salt are a feature of SS hypertension.

It is well-known that SS hypertension is associated with more severe target organ damage than SR hypertension and that this may be explained by worse endothelial dysfunction in the former (Franco and Oparil, 2006). It is therefore highly conceivable that ET-1-dependent and IR-dependent endothelial dysfunction are important determinants of the early and severe target organ damage of SS hypertension, and thus explain the poor prognosis associated with this phenotype.

### Adipokines in Insulin Resistance and Hypertension

As a key element of metabolic syndrome, obesity closely relates with both hypertension and insulin resistance. Increased adiposity is proposed to contribute to both blood pressure and glucose homeostasis through secretion of various adipokines. One such is chemerin, an adipocyte-derived factor responsible for the development and differentiation of adipocytes (Goralski et al., 2007). Chemerin has vasoconstrictive effects through its actions on vascular smooth muscle (Ferland et al., 2017) and endothelium (Lobato et al., 2012) in both human and animal vasculature (Kennedy et al., 2016) and decrease in whole body chemerin by chemerin antisense oligonucleotides reduces blood pressure in normal-fed rats (Ferland et al., 2018) as well as in Dahl salt-sensitive (DS) rats fed high-salt and high fat diets (Ferland et al., 2019). In humans, chemerin has been found to associate with higher risk of hypertension in a large population-based study including 3,986 subjects (Zylla et al., 2017).

In addition, chemerin regulates glucose metabolism and its actions in skeletal and cardiac muscles have been linked with insulin resistance in several studies (Sell et al., 2009; Becker et al., 2010; Zhang et al., 2014). Rodent models of obesity/diabetes demonstrated significantly elevated serum levels of chemerin; exogenous chemerin administration further exacerbated glucose intolerance (Ernst et al., 2010). Nevertheless, human studies have not shown a consistent relationship between insulin sensitivity and serum chemerin levels and its exact role in whole body insulin resistance remains controversial (Takahashi et al., 2008; Alfadda et al., 2012; Helfer and Wu, 2018; Karczewska-Kupczewska et al., 2020).

Another factor produced by adipose tissue that has overlapping roles in obesity, insulin resistance, and hypertension is leptin. Data from human as well as animal studies suggest that leptin plays a major role in the neurohormonal mechanisms of obesity-induced hypertension (Simonds et al., 2014). Obese subjects display high circulating levels of leptin and an insensitivity to the anorexigenic effects of exogeneous leptin, known as leptin resistance (Izquierdo et al., 2019). Leptin is proposed to affect blood pressure through its sympathetic activity and leptin levels directly associated with blood pressure changes in rodent models of obesity, which is not seen in models of leptin deficiency. Humans with loss-of-function mutation in leptin or leptin receptor have been found to be protected from hypertension despite obesity (Simonds et al., 2014), nevertheless, exogenous leptin administration does not increase blood pressure in leptin deficient states including congenital leptin deficiency and lipodystrophy (Brown et al., 2015). Importantly, obesity secondary to leptin resistance was found to result in increased salt sensitive blood pressure response to high salt in SHHF rat, a model of spontaneous hypertension. The increase in salt sensitivity was driven by endothelin and was obliterated by bosentan (Radin et al., 2003). In another study, high salt diet has been demonstrated to cause leptin resistance, obesity as well as high blood pressure and insulin resistance in mice, further implying a link between leptin resistance, high salt-associated hypertension, and insulin resistance (Lanaspa et al., 2018).

Leptin also has important pro-inflammatory effects, which may play a role in insulin resistance and hypertension. Leptin activates monocytes to produce inflammatory cytokines such as tumor necrosis factor (TNF)-α, IL-6, and IL-12(Gainsford et al., 1996), while suppressing the anti-inflammatory Th2 response (Fernández-Riejos et al., 2010). The chronic inflammatory state observed in high adiposity, whether partially driven by leptin or not, seems to play a major role in the interplay between insulin resistance and hypertension.

### Immunity in Obesity and IR

Obesity is characterized by high levels of plasma inflammatory cytokines (Villarreal-Calderón et al., 2019) and a low-grade inflammatory state in visceral adipose tissue, which is a critical contributor to the development of insulin resistance (Winer and Winer, 2012). The innate immune system plays an important role in the proinflammatory state seen in obesity (Patel et al., 2013). Macrophages of the inflamed adipose tissue are polarized to the M1 state with enhanced antigen-presenting and proinflammatory cytokine-producing properties, to the detriment of M2 macrophages that produce anti-inflammatory mediators such as IL-10 and TGF-β (Orliaguet et al., 2020). M1 production of TNF-α, IL-1β, and IL-6, IL-12, and iNOS activates serine kinases that phosphorylate IRS proteins and insulin receptors, thus hindering insulin signaling (Mantovani et al., 2002; Olefsky and Glass, 2010). This paracrine process in the adipose tissue has local and also systemic effects due to leakage of cytokines into circulation, which can be detected with plasma measurements (Olefsky and Glass, 2010).

The adaptive immune system is also activated in obesity. T cells accumulate in the adipose tissue and interact with macrophages to enhance the inflammatory response (Nishimura et al., 2009). Indeed, obesity and insulin resistance are associated with predominance of CD8^+^ and CD4^+^ Th1 T cells with IFN-γ secretion in visceral adipose tissue that promotes the M1 polarization of macrophages described above (Winer and Winer, 2012). In mice fed a high-fat diet, depletion of CD8^+^ T cells reduces local adipose tissue inflammation and insulin resistance, whereas conversely, transfer of CD8^+^ cells into CD8 deficient mice induces insulin resistance (Nishimura et al., 2009).

## Immunity in Hypertension

Hypertension is also strongly associated with inflammation. T cells and macrophages infiltrate the perivascular space and kidneys in animal models of hypertension (Kirabo et al., 2014; Caillon et al., 2017) and relate to hypertensive end organ damage. Mice lacking T cells or the T cell cytokine IL-17A are protected from angiotensin II-induced hypertension and related vascular dysfunction (Crowley et al., 2010; Madhur et al., 2010). Moreover, immunosuppression has antihypertensive effects in animal studies (McMaster et al., 2015).

Hypertension is associated with T cell clonal expansion, a hallmark of antigen-presentation found in various immune pathologies including autoimmune disease (Acha-Orbea et al., 1998), atherosclerosis (Paulsson et al., 2000), and obesity (Yang et al., 2010). By examining T cell receptor (TCR) usage, we showed that accumulation of an oligoclonal CD8^+^ T cell population in the kidney contributes to hypertension by inducing endothelial dysfunction and vascular rarefaction as well as sodium and volume retention (Trott et al., 2014). More recently, we found that the CD8^+^ T cell populations in the adipose tissue of hypertensive high-fat fed mice are more clonal and demonstrate enrichment for positively charged amino acids, particularly arginine, which is a characteristic previously reported in TCRs associated with obesity and insulin resistance (McDonnell et al., 2018). Furthermore, two of the TCR clonotypes found in abundance in adipose tissue of high-fat fed mice were previously identified diabetogenic clonotypes (McDonnell et al., 2018).

In multiple hypertensive models, we showed that hypertension is associated with production of isolevuglandins (IsoLGs; alternatively named Isoketals or γ-ketoaldehydes) in murine dendritic cells (DCs) and human monocytes (Figure 2). IsoLGs are highly reactive products of lipid peroxidation and adduct to lysine residues on proteins, leading to alteration of protein function and formation of neoantigens (Kirabo et al., 2014). These immunogenic proteins lead to T cell activation and hypertension (Kirabo et al., 2014), and contribute to the oligoclonal T cell expansion in the adipose tissue of high-fat induced obesity (McDonnell et al., 2018). These protein adducts, owing to their negative charge (McDonnell et al., 2018) particularly associate with TCRs found in obese adipose tissue, which are enriched with positively charged amino acids. IsoLGs represent a potential interventional target, since administration of isoLG scavengers prevents the inflammatory responses and the development of hypertension (Kirabo et al., 2014).

Moreover, we reported that elevated interstitial Na^+^ is a potent stimulus for IsoLG-adduct formation in murine DCs. Na^+^ enters DCs through amiloride-sensitive ENaC channels and promotes calcium (Ca^2+^) entry into the cell *via* the Na^+^/Ca^2+^ exchanger. Increased intracellular Ca2^+^ activates protein kinase C (PKC), which phosphorylates and activates the NADPH oxidase. The increased superoxide and ROS formation by NADPH oxidase promote IsoLG formation and subsequent DC activation (Barbaro et al., 2017). These high-salt treated DCs produce IL-1β and induce T cell production of pro-hypertensive cytokines IL-17A and IFN-γ (Barbaro et al., 2017). Other studies of mice have also shown increased circulating IL-17 levels in response to high salt diet, leading to endothelial dysfunction (Faraco et al., 2018). These findings indicate that elevated tissue Na^+^ may contribute to the development of hypertension and salt sensitivity through immune cell activation. Indeed, previous studies suggested altered regulation of the hyperosmolar Na^+^ storage in tissue in the pathogenesis of salt sensitivity (Laffer et al., 2016). Whether differences in tissue Na^+^ or differences in the immune response to tissue Na^+^ play a role in the salt sensitive phenotype is yet to be discovered.

The relationship between the immune system and interstitial Na^+^ stores is complex. It is pretty clear that interstitial Na^+^ storage triggers immune system activation. Alternatively, the immune system regulates interstitial Na^+^ storage. As discussed earlier, interstitial Na^+^ storage in tissue, primarily in muscle and skin, may be an important buffer system for high salt intake that may determine SSBP. Innate immune cells have been suggested to play a role in the regulation of the amount of Na^+^ that can be stored in the interstitium, which in turn may activate adaptive immune cells through ENaC mediated Na^+^ entry into antigen presenting cells (APCs) as described above. The mononuclear phagocyte system (MPS) and macrophages have been shown to regulate tissue Na^+^ accumulation through modulating interstitial Na^+^ clearance. The high salt diet-induced increase in skin Na^+^ relates with hyperplasia of the lymphatic capillary network, through activation of TonEBP and consequent secretion of VEGF-C by macrophages (Machnik et al., 2009). This lymphangiogenesis provides a buffer for extra sodium and water in tissue as shown by the fact that either depletion of MPS or inhibition of VEGF action augmented interstitial hypertonic water retention and salt-induced blood pressure elevation (Machnik et al., 2009). The MPS/TonEBP/VEGF-C pathway belongs to the physiological anti-inflammatory phenotype of M2 subtype of macrophage polarization (Machnik et al., 2010). Shifting the balance of macrophage polarization into the pro-inflammatory M1 subtype associated with insulin resistant states, may disrupt MPS modulation of interstitial Na^+^. This most likely converted the salt resistant rats into a salt sensitive phenotype (Machnik et al., 2010) in the experiments above.

## Role of the Inflammasome

The inflammatory responses seen in both hypertension and obesity induced insulin resistance include inflammasome activation. NOD-like receptor family pyrin domain containing 3 (NLRP3) inflammasome, is a member of the nucleotide-binding oligomerization domain leucine-rich repeat (NLR) PRR family and drives sterile inflammation in response to damaged cell derived “danger-associated molecular patterns,” leading to caspase-1 activation and subsequent IL-1β and IL-18 secretion (Chen and Nunez, 2010; Van Tassell et al., 2013; Lamkanfi and Dixit, 2014).

NLRP3 has also been suggested to produce inflammatory responses in adipose tissue, since they can be triggered by ceramides, a product of fatty acid metabolism, as well as oxidized LDL and cholesterol (Duewell et al., 2010). The common end point of NLRP3 inflammasome activation is a chronic systemic inflammatory state because the locally secreted inflammatory cytokines are released into blood with subsequent development of global insulin resistance (Xu et al., 2003; Zhou et al., 2014). In animal models, elimination of NLRP3 inflammasome protects from high-fat induced insulin resistance, while in obese diabetic individuals, adipose tissue expression of NLRP3 inversely correlates with the increase in insulin sensitivity after weight loss (Vandanmagsar et al., 2011). Analysis of UKBiobank revealed that individuals with a gene variant increasing NLRP3 mRNA expression was associated with higher prevalence of diabetes (Schunk et al., 2021).

Hypertension has also been related with the activation of NLRP3 inflammasome. IL-1β and IL-18, the main products of NLRP3 activation, are elevated in plasma and monocytes of hypertensive individuals (Dörffel et al., 1999; Li et al., 2005; Rabkin, 2009) and associate with renal and vascular dysfunction (De Miguel et al., 2021). Hypertensive patients also have high concentrations of NF-κB, a crucial intracellular trigger of NLRP3 activation, in tissue and inflammatory cells (De Miguel et al., 2021). NF-κB inhibition ameliorates hypertension and prevents hypertension-induced organ damage in mice (Zambom et al., 2019). NF-κB may also play a role in the interplay between salt sensitivity and insulin resistance, since the association between these two traits in Dahl salt sensitive rats was partially NFkB dependent. Pyrrolidine dithiocarbamate (PDTC), an inhibitor of NFκB, significantly improved blood pressure as well as insulin sensitivity and insulin mediated vasorelaxation in this salt sensitive rat model (Zhou et al., 2010). Indeed, NLRP3 (Sun et al., 2017) and NALP3 (Fu et al., 2018) inflammasomes mediate endothelial dysfunction, main contributors to both salt sensitivity and insulin resistance as proposed earlier. Moreover, eNOS and NO pathways, which are disrupted in salt sensitivity, are in part regulated by the NLRP3 inflammasome (Sogawa et al., 2018). Importantly, NLRP3 inflammasome is activated by NADPH oxidase and ROS (Abais et al., 2014; Cui et al., 2014; Banoth and Cassel, 2018), which are crucial mediators of IsoLG formation.

### Immunity and the Link Between SSBP and IR

While inflammation has been shown to separately contribute to the pathogenesis of both insulin resistance and salt sensitive hypertension, is not known whether it has a causal role in the link between these two traits (Figure 3). Animal models extensively used as a paradigm for salt-sensitive hypertension in humans provide some evidence in this regard. In DS rats, inflammation and oxidative stress were found to associate with both hypertension and insulin resistance (Zhou et al., 2010). Furthermore, treatment with tempol, an intracellular antioxidant, improved inflammatory markers, vascular insulin signaling, endothelium-dependent relaxation, and insulin sensitivity. Similarly, the angiotensin receptor blocker candesartan, a commonly used antihypertensive, also improved inflammatory markers and insulin sensitivity (Zhou et al., 2009).

The potential role of inflammation in the pathogenesis of SS may also have further implications regarding the increased cardiovascular risk in individuals with SS. Considering that pro-inflammatory status is consistently associated with increased risk of cardiovascular disease (Sorriento and Iaccarino, 2019), it is plausible that inflammatory activation related to SS development may also link with higher cardiovascular risk. Furthermore, if elevated, interstitial Na^+^ may indeed lead to a systemic immune activation through infiltration of T cells in various organs, an altered tissue Na^+^ storage may potentially predispose the salt sensitive population to poor cardiovascular outcomes.

### PPARγ, Insulin Resistance, and Immunity

The peroxisome proliferator-activated receptors are a nuclear receptor superfamily of ligand-inducible transcription factors found in humans and includes three subtypes: PPARα, PPARβ/δ, and peroxisome proliferator-activated receptor γ (PPARγ; Ahmadian et al., 2013). PPARγ is well-known as the master regulator of adipogenesis and lipid metabolism and is predominantly expressed in white and brown adipose tissue. PPARγ is bound and activated by fatty acids and their derivatives endogenously and by thiazolidinediones (TZDs), highly specific synthetic ligands, exogenously. PPARγ activation has a robust insulin sensitizing effect, making TZDs potent insulin sensitizers and highly effective oral medications for type 2 diabetes, although, their clinical use is limited by major adverse effects including weight gain, fluid retention, and osteoporosis (Ahmadian et al., 2013).

PPARγ acts through various gene networks to modulate glucose homeostasis. Its activation is directly related with increased expression of glucose transporter type 4, while also controlling the expression of many factors that affect insulin sensitivity, such as adiponectin, resistin, leptin, and TNF-α. Therefore, PPARγ agonists’ insulin sensitizing effects can be explained by various mechanisms including modulation of cellular glucose update, hepatic glucose release, systemic inflammation, or food intake (Iwaki et al., 2003; Tomaru et al., 2009; Ahmadian et al., 2013). Indeed, PPARγ’s regulatory roles are not limited to adipocytes. PPARγ also has crucial functions in immune cells, especially in macrophages and APCs (Tontonoz et al., 1998; Wahli and Michalik, 2012). PPARγ regulates lipid metabolism and exerts anti-inflammatory effects in macrophages, including polarization into the anti-inflammatory M2 subtype (Odegaard et al., 2007). Mice with macrophage specific deletion of PPARγ exhibit disruption of M2 macrophage activation, and development of diet induced obesity and whole-body insulin resistance (Odegaard et al., 2007). In turn, evidence suggest that TZDs downregulate the expression of M1 mediators (Xu et al., 2016). Moreover, PPARγ also regulates the accumulation and function of regulator T cells in the visceral adipose tissue and expression of PPARγ in this unique T cell population is essential for the complete insulin-sensitizing activity of TZDs in obese mice (Cipolletta et al., 2012). PPARγ-axis dependent insulin signaling has also shown to drive state transition between adipose regulatory T cell subsets (Li et al., 2021). PPARγ agonists have also been reported to suppress the progression of atherosclerosis and aortic aneurysmal changes by reducing the expression of TNF-α and other markers of inflammation (Zinn et al., 2008; Motoki et al., 2015). These anti-inflammatory actions of PPARγ can potentially explain some of its beneficial effects on insulin sensitivity as well as blood pressure (Figure 4).

### PPARγ and Hypertension

Compelling evidence suggest that PPARγ is also an important regulator of blood pressure. Various mutations in PPARγ, causing loss of function, have been shown to strongly associate not only with insulin resistance and diabetes but also with severe hypertension (Barroso et al., 1999; Fang et al., 2021). In addition to these rare mutations, common variants of PPARγ may also play a role. For example, in a meta-analysis of more than 5,500 Asian hypertensives (Zhang et al., 2019), the P12A polymorphism was associated with the risk of hypertension. Furthermore, treatment with PPARγ agonists is associated with decreased blood pressure (Nolan et al., 1994).

A well-known side effect of TZD is fluid retention. TZDs increase renal sodium reabsorption without modulation of RAS system, in people with diabetes and hypertension (Zanchi et al., 2010) and in healthy subjects (Zanchi et al., 2004). Despite this, large scale clinical studies consistently show that TZDs, including pioglitazone (Dormandy et al., 2005; Yoshii et al., 2014), rosiglitazone (Komajda et al., 2008), and troglitazone (Nolan et al., 1994), lead to modest decreases in blood pressure (Qayyum and Adomaityte, 2006). Various cardiovascular actions of TZD could explain its effect on blood pressure, including its anti-inflammatory and endothelium-mediated vasodilatory actions. PPARγ activation upregulates eNOS expression and availability of vascular NO (Sartori-Valinotti et al., 2010; Nagao and Yamaguchi, 2012), while it downregulates the synthesis of vasoconstrictor endothelin-1 (Satoh et al., 1999; Kang et al., 2017) and the expression of Angiotensin II type 1 receptor [AT (1)-R], thus promoting vasodilation in the vascular smooth muscle (Ketsawatsomkron et al., 2012; Pelham et al., 2012). Mice lacking endothelial-specific PPARγ have deficient NO production and increased oxidative stress markers (Kleinhenz et al., 2009), and show augmented Ang-II induced endothelial dysfunction, a response dependent on superoxide, NADPH oxidase, and Rho kinase (Silva et al., 2017). PPARγ further inhibits pro-inflammatory gene expression in vascular smooth muscle through inhibition of NF-κB activity (Mukohda et al., 2017) and protects against IL-1β-induced oxidative stress in endothelium (Mukohda et al., 2016).

Considering the impaired vasodilatory response observed in salt sensitive patients and the beneficial effects of PPARγ on the vasodilatory system, PPARγ agonists would be expected to ameliorate salt sensitivity of blood pressure (SSBP). Indeed, despite increased sodium retention, clinical studies demonstrated that TZD abolished the blood pressure response to salt in salt-sensitive individuals (Zanchi et al., 2010). In animal models, TZDs also attenuated salt sensitivity, a result observed along with increased NO availability and decreased renal macrophage infiltration and inflammation (Sartori-Valinotti et al., 2010). Furthermore, mice models of PPARγ impairment develop salt sensitive hypertension. Salt sensitive humans and animals demonstrate decreased renal blood flow and increased renal vascular resistance, and these abnormalities are attenuated by TZDs (Fink et al., 1980; Campese et al., 1991; Wu et al., 2021). Mice with dominant negative mutation of PPARγ in vascular smooth muscle start showing salt-induced impairment of vasodilation along with blunted NO responsiveness after 3 days of high salt diet, and this is followed by the development of salt sensitive hypertension (Wu et al., 2021). Regarding its actions to increase insulin sensitivity, PPARγ agonists can also counteract the effects of insulin resistance on blood pressure. In insulin resistant fatty rat models, rosiglitazone treatment prevents the development of hypertension and partially restores the vasodilatory effects of insulin, thus ameliorating endothelial dysfunction associated with insulin resistance (Walker et al., 1999). Therefore, mounting evidence points to PPARγ as a common regulator of both glucose and blood pressure homeostasis. As numerous mutations of PPARγ have been shown to result in both severe insulin resistance and hypertension, deficiency of physiologic PPARγ activation may be a pathogenetic factor linking insulin resistance and salt sensitive hypertension, a question that remains to be answered in definitive manner.

### Oxidative Metabolites, IR, and SSBP

Methylglyoxal (MGO), a metabolite of the glycolysis pathway that is increased in diabetes, has been proposed to contribute to the development of salt sensitivity as well as insulin resistance through oxidative stress and advanced glycation endproducts (AGE; Guo et al., 2009). Increased levels of MGO along with increased levels of ROS have been found in vascular tissue of hypertensive animals (Chang et al., 2005; Jia and Wu, 2007) and in plasma of diabetic patients. High fructose induced accumulation of endogenous MGO in plasma and tissue induces the development of hypertension and insulin resistance in rats (Wang et al., 2008).

The mechanisms by which MGO produces insulin resistance are multiple. Accumulation of endogenous MGO reduces IRS-1/PI3K association and alters PI3K activity in adipose tissue, leading to decreased insulin stimulated glucose uptake and insulin resistance (Jia and Wu, 2007). Other studies have also shown that MGO disrupts insulin signaling pathways by formation of AGEs and ROS (Unoki et al., 2007) and by direct binding to IRS1 protein. This inhibits IRS1 association with other proteins in cultured muscle cells and in adipose tissue of fructose-induced hypertensive rats independent of formation of ROS (Riboulet-Chavey et al., 2006). Furthermore, MGO directly produces a dose-dependent decrease in adipose tissue capillarization and blood flow, which associate with systemic and muscle insulin resistance (Rodrigues et al., 2017). Finally, MGO also alters insulin structure by binding to its arginine residue and decreasing its activity (Jia et al., 2006).

In addition to its role in the pathogenesis of insulin resistance, MGO contributes to the development of salt sensitivity of blood pressure by mechanisms not yet fully understood. In normotensive rats, MGO treatment induces both the development of insulin resistance and salt sensitivity. Although MGO or salt, given separately did not induce hypertension, co-administration of both significantly increased blood pressure. N-acetyl cysteine, a MGO scavenger, or an AGE inhibitor completely improved MGO-induced insulin resistance in this model (Guo et al., 2009), whereas metformin improved both, the increase in endogenous MGO and in blood pressure observed in a high fructose diet model (Wang et al., 2008).

## Conclusion

Salt sensitivity of blood pressure, a significant cardiovascular risk factor, strongly associates with insulin resistance. Salt sensitivity and insulin resistance share several pathogenetic factors. They include vascular dysfunction, particularly caused by endothelin-1 overproduction, and immune activation primarily driven by CD8^+^ T cells action. The transcription factor PPARγ also modulates insulin sensitivity and hypertension through its anti-inflammatory and vasodilatory actions. Impairment of PPARγ action results in insulin resistance and hypertension in both animal models and humans, and perhaps is a common denominator linking insulin resistance and salt sensitive hypertension in the population. The oxidative metabolite of the glycolysis pathway MGO, also contributes to the development of both salt sensitivity and insulin resistance through yet to be explored mechanisms. The presence of these shared pathways underlying the mechanisms of both, insulin resistance and salt sensitivity of blood pressure, is very suggestive of a possible causal bidirectional relationship between these two cardiovascular risk factors, a contention that remains to be proven in definitive manner.

## Author Contributions

LE wrote the draft. FE, CL, and AK revised and approved the manuscript. AK obtained funding for the manuscript. All authors contributed to the article and approved the submitted version.

## Funding

This study was supported by the Vanderbilt CTSA grant UL1TR002243 from NCATS/NIH and the National Institutes of Health grants K01HL13049, R03HL155041, and R01HL144941 to AK.

## Conflict of Interest

The authors declare that the research was conducted in the absence of any commercial or financial relationships that could be construed as a potential conflict of interest.

## Publisher’s Note

All claims expressed in this article are solely those of the authors and do not necessarily represent those of their affiliated organizations, or those of the publisher, the editors and the reviewers. Any product that may be evaluated in this article, or claim that may be made by its manufacturer, is not guaranteed or endorsed by the publisher.
